# Stereotactic radiotherapy or metastasectomy for oligometastatic esophagogastric cancer: A nationwide population-based cohort study

**DOI:** 10.1016/j.ctro.2022.08.012

**Published:** 2022-08-24

**Authors:** Tiuri E. Kroese, Nikita K.N. Jorritsma, Hanneke W.M. van Laarhoven, Rob H.A. Verhoeven, Stella Mook, Nadia Haj Mohammad, Jelle P. Ruurda, Peter S.N. van Rossum, Richard van Hillegersberg

**Affiliations:** a Department of Surgery, University Medical Center Utrecht, Utrecht University, Utrecht, The Netherlands; b Department of Radiation Oncology, University Medical Center Utrecht, Utrecht University, Utrecht, The Netherlands; c Department of Medical Oncology, Amsterdam University Medical Center, University of Amsterdam, Amsterdam, The Netherlands; d Department of Research & Development, Netherlands Comprehensive Cancer Organization (IKNL), Utrecht, The Netherlands; e Department of Medical Oncology, University Medical Center Utrecht, Utrecht University, Utrecht, The Netherlands

**Keywords:** Esophageal cancer, Gastric cancer, Metastasectomy, Stereotactic radiation therapy, Oligometastasis, OS, Overall survival, OMD, Oligometastatic disease, SBRT, Stereotactic body radiotherapy, NCR, Netherlands Cancer Registry, RCT, Randomized controlled trial, HR, Hazard ratio, SD, Standard deviation, IQR, Interquartile range

## Abstract

**Background and purpose:**

This nationwide population-based study analyzed the outcomes of local treatment (i.e. stereotactic body radiotherapy [SBRT] or metastasectomy) or systemic therapy for oligometastatic disease (OMD) in patients with esophagogastric cancer in The Netherlands.

**Materials and methods:**

Between 2015 and 2016, all patients in The Netherlands with esophagogastric cancer and synchronous or metachronous OMD were eligible for inclusion. Patients who underwent local treatment of OMD (SBRT or metastasectomy) and/or systemic therapy were included. OMD was defined as distant metastases in 1 organ or 1 extra-regional lymph node region. The primary outcomes were overall survival (OS) and independent prognostic factors for OS. OS was calculated from diagnosis of OMD. Prognostic factors for OS were analyzed using a multivariable Cox proportional hazard model.

**Results:**

A total of 594 patients were included, of whom 83 underwent local treatment for OMD alone, 22 local treatment plus systemic therapy, and 489 systemic therapy alone. Median OS after local treatment for OMD alone was 16.0 months, local treatment plus systemic therapy 22.7 months, and after systemic therapy alone 8.5 months. Improved OS was independently associated with local treatment for OMD alone or combined with systemic therapy as compared with systemic therapy alone (hazard ratio [HR] 0.52, 95% CI: 0.31–0.90 and HR 0.42, 95% CI: 0.22-0.82, respectively) and a controlled primary tumor(HR 0.48, 95% CI: 0.27–0.86). Worse OS was independently associated with worse performance scores (HR 1.41, 95%: 1.32-1.75), poorly or undiffertumor as compared with good or moderadifferentiated tumor (HR 1.37, 95% CI: 1.06-1.76), and peritoneal as compared with lymph mode metastases (HR 1.39, 95% CI: 1.00-1.93).

**Conclusion:**

Local treatment of OMD alone or combined with systemic therapy was independently associated with improved OS as compared with systemic therapy alone in this population-based cohort study in The Netherlands. Randomized controlled trials are warranted to confirm these results.

## Highlights

• This nationwide study included patients with esophagogastric cancer and OMD.
• Local treatment of OMD was associated with improved OS.
• Local treatment of OMD plus systemic therapy was associated with better OS.
• Randomized trials are warranted to conform results.

## Graphical abstract

**Figure:**
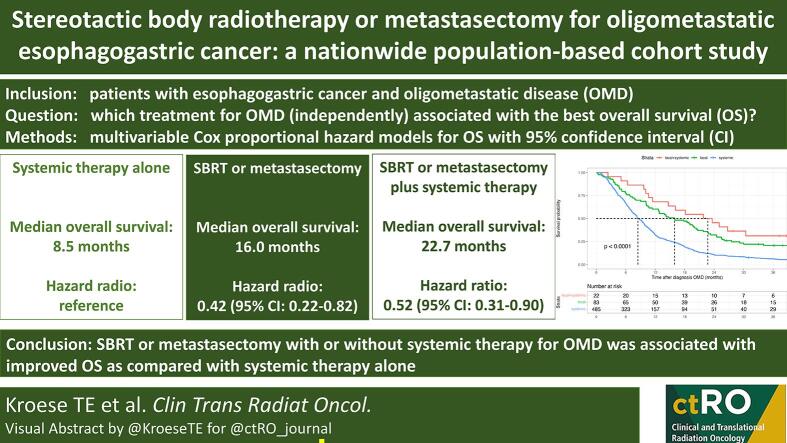

## Introduction

Gastric and esophageal cancer are the 5th and 7th most common cancers worldwide and the incidence of esophageal cancer is rapidly rising [1]. Approximately 30–50 % of patients with esophagogastric cancer (i.e. esophageal or gastric cancer) have metastatic disease at the time of initial diagnosis (i.e. synchronous) [2]. In addition, >30 % of patients develop metastatic disease during follow-up after initial primary tumor treatment with curative intent (i.e. metachronous) [3], [4]. Patients with metastatic esophagogastric cancer have a poor prognosis, with a median overall survival (OS) between 3 and 9 months [4], [5], [6], and are usually treated with systemic therapy or best supportive care [7], [8], [9], [10].

In a small portion of metastatic patients, distant metastases are limited in number and distribution, so-called oligometastatic disease (OMD) [11]. OMD reflects a disease state between locoregional and widespread metastatic disease [11]. Randomized controlled trials (RCTs) have shown that local treatment (e.g. metastasectomy or stereotactic body radiotherapy [SBRT]) improves OS as compared with systemic therapy alone in patients with breast, prostate, colorectal, or lung cancer [12], [13]. For esophagogastric cancer, phase II trials have suggested improved OS after local treatment of OMD [14], [15], which is currently being investigated in RCTs [16], [17], [18].

However, the applicability and generalizability of the currently available data from the literature is unclear since clinical trial results cannot always be reproduced in the real-world setting due to strict selection criteria [19]. Therefore, real-world population-based data are a valuable addition to trial results because they deepen the understanding of the outcome of therapies in patients encountered on a day-to-day basis, making results better interpretable in clinical practice [20]. Furthermore, population-based studies enable us to analyze a relatively large population considering the proportion of patients receiving local treatment for OMD is relatively small [21]. Finally, the adoption of local treatment of OMD varies and knowledge on outcomes on a population-based level is currently lacking. Therefore, this study aimed to determine OS and independent prognostic factors for OS after local treatment or systemic therapy for OMD in patients with esophagogastric cancer on a nationwide population-based level.

## Methods and materials

### Study design

This study included patients registered in the Netherlands Cancer Registry (NCR). The NCR is the only national oncological registry in The Netherlands and provides cancer statistics among all 17.4 million residents. According to the Central Committee on Research involving Human Subjects, this study did not need approval by an institutional review board in The Netherlands. The study was approved by the Privacy Review Board of the Netherlands Cancer Registry and the scientific committee of the Dutch Upper GI Cancer Group (DUCG). The study was reported according to the guidelines of The Strengthening the Reporting of Observational Studies in Epidemiology (STROBE) Statement (Supplementary File A) [22].

### Patient inclusion

Consecutive patients with synchronous or metachronous metastatic esophagogastric cancer were identified from the NCR between 2015 and 2016 (i.e. according to UICC/AJCC 7th edition [18] as Tx-4b, Nx-N3, M1 and according to ICD-10 [23] as 15.3–15.5, 15.8, 15.9, and 16.0–16.9). The years 2015 and 2016 were selected because the NCR registered additional data on metachronous metastases for these years only. OMD was defined as distant metastases in 1 organ or 1 extra-regional lymph node region comparable with a recent systemic review on definitions of oligometastatic esophagogastric cancer in current literature [24]. OMD was not defined by a maximum number of lesions per organ/extra-regional lymph node station because this was not recorded by the NCR. Patients undergoing local treatment of OMD (i.e. SBRT or metastasectomy) or systemic therapy were included. SBRT was defined as radiotherapy according to one of the following radiotherapy schemes: ≥10 Gy per fraction with ≥1 fraction, ≥5 Gy per fraction with ≤12 fractions, or ≥7 Gy per fraction with ≤5 fractions. All other radiotherapy schemes were considered palliative radiotherapy. Patients undergoing palliative radiotherapy were not included. Metastasectomy was defined as surgery, which could include radiofrequency ablation.

### Variables

From the NCR patient characteristics were extracted, including sex, age, and WHO performance score. WHO performance score was determined at the time of treatment of OMD. Collected disease characteristics included clinical and pathological disease stage (according to UICC 7th edition [25], histology, tumor differentiation grade, and morphology (i.e. signet ring cell carcinoma). The OMD state was categorized into synchronous or metachronous (defined as before or after completion of primary tumor treatment, respectively [26]. The location of OMD lesions was categorized into a distant organ (e.g. lung, liver, or brain), an extra-regional lymph node region (i.e. head and neck, intra-thoracic, intra-abdominal, axilla, pelvic, multiple locations, or not specified [23], or peritoneal (i.e. peritoneum, ovary, or omentum). Finally, treatment characteristics were extracted, including treatment of the primary tumor and OMD and the type of hospital where this treatment was performed. Hospitals were categorized into ‘academic’, or ‘non-academic’.

### Treatment of primary tumor and oligometastasis

The primary tumor was considered controlled in patients who underwent primary tumor resection or definitive chemoradiotherapy (radiotherapy to dose ≥50 Gy with concurrent chemotherapy) without evidence of locoregional recurrence at the time of OMD detection. Treatment of OMD was categorized into 1) local treatment alone (i.e. SBRT and/or metastasectomy); 2) local treatment plus systemic therapy (i.e. chemotherapy or targeted therapy); 3) systemic therapy alone. The administration of systemic therapy was divided into before or after local treatment of OMD. The first-line systemic therapy regimen administrated after the diagnosis of current OMD was analyzed (i.e. second-line systemic therapy for recurrent or progressive disease was not analyzed).

### Outcome

The primary outcomes of this study were OS and prognostic factors for OS. OS was defined as the time interval between the diagnosis of OMD and death or end of follow-up. Vital status was obtained through annual linkage with the municipal population registers and was last updated on January 31, 2021. Prognostic factors for OS were expressed using hazard ratios (HRs) with 95% confidence intervals (CIs). Kaplan-Meier curves were constructed for OS and independent prognostic factors for OS and were compared using log-rank test.

### Statistical analysis

Parametric data were presented as mean with standard deviation (SD) and were compared using Student’s *T* test. Non-parametric data were presented as median with interquartile range (IQR) and compared using Mann Whitney *U* test. Categorical data were presented as frequencies with proportions and compared using Fisher’s exact test. Factors previously identified in literature [27] as prognostic factors for OS in metastatic esophagogastric cancer were entered into univariable and multivariable Cox proportional hazard model, which included WHO performance score (WHO 0 versus >0 versus missing) [28], tumor differentiation grade (well/moderate versus poorly/undifferentiated versus missing) [29], histology (adenocarcinoma versus squamous cell carcinoma) [28], OMD state (synchronous versus metachronous) [30], primary tumor treatment status (controlled versus not controlled) [31], treatment of OMD (local treatment versus local treatment plus systemic therapy) [32], and location of OMD (extra-regional lymph node versus peritoneum versus organ) [14]. The disease-free interval for metachronous OMD was defined as the time interval between the diagnosis of the primary tumor and OMD. Complete-case analyses were performed. The median follow-up time was estimated using the reverse Kaplan-Meier estimator (i.e. reverse event indicator). Data were analyzed using R for Windows, version 3.6.3. A two-sided p-value < 0.05 was considered statistically significant.

## Results

**Fig. 1:**
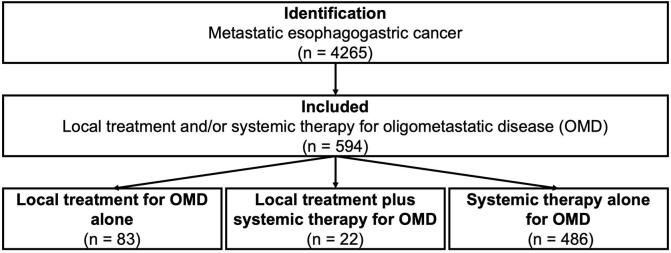
Flowchart of patient inclusion.

Between 2015 and 2016, 4265 patients with synchronous or metachronous metastatic esophagogastric cancer were identified from the NCR, of whom 594 patients who underwent local treatment or systemic therapy for OMD were included. First, the 105 patients undergoing local treatment for OMD with or without systemic therapy will be described. Subsequently, the 489 patients undergoing systemic therapy alone for OMD (Fig. 1).

The 105 included patients were generally male (71%) with a mean age of 64 years (SD: ±8) and mostly had a WHO performance score of 0–1 at the time of treatment (62%). The primary tumors were predominantly adenocarcinomas (80%) located in the distal third of the esophagus (57%). The predominant clinical tumor stage was cT3 (66%) and nodal stage cN1 (45%). For patients who underwent primary tumor resection (n = 74), the predominant pathological tumor stage was pT3 (45%) and nodal stage pN0 (45%).

Most patients had metachronous OMD (62%, i.e. OMD detected after primary tumor treatment). OMD was located in 1 distant organ (79%), 1 extra-regional lymph node region (12%), or the peritoneum (9%). The median disease-free interval for metachronous OMD was 17 months (IQR: 14–24) after diagnosis of the primary tumor. OMD was confirmed with pathological assessment (71%) or repeated follow-up imaging (29 %, Table 1).

**Table 1:** Patient and tumor characteristics of included patients.

Factor	Local +/− systemic therapy (n = 105)	Systemic therapy only (n = 489)	P-value
**Mean age in years (±SD)**	64 (±8)	64 (±10)	0.894

**Sex**			0.460
Male	75 (71 %)	369 (75 %)	
Female	30 (29 %)	120 (25 %)	

**WHO performance score**			<0.001
0	35 (33 %)	119 (24 %)	
1	27 (29 %)	165 (34 %)	
>1	6 (5 %)	53 (11 %)	
Missing	37 (33 %)	152 (31 %)	

**Location of the primary tumor**			<0.001
Upper or middle third esophagus	14 (13 %)	51 (10 %)	
Lower third esophagus	60 (57 %)	187 (38 %)	
Esophagus not specified	2 (2 %)	14 (3 %)	
Gastroesophageal junction/cardia	13 (12 %)	80 (16 %)	
Stomach	16 (15 %)	157 (32 %)	

**Clinical tumor stage**			<0.001
cT1b or cT2	25 (24 %)	169 (35 %)	
cT3 or cT4	74 (70 %)	168 (35 %)	
Missing	5 (5 %)	102 (21 %)	

**Clinical nodal stage**			0.124
cN0	30 (29 %)	121 (25 %)	
cN1	48 (46 %)	165 (34 %)	
cN2 or cN3	26 (25 %)	168 (34 %)	
Missing	1 (1 %)	28 (6 %)	

Pathological tumor stage*	**Total (n = 74)**	**Total (n = 89)**	0.349
pT0	12 (16 %)	8 (9 %)	
pT1 or pT2	25 (33 %)	37 (42 %)	
pT3 or pT4	36 (48 %)	42 (47 %)	
Missing	1 (1 %)	2 (2 %)	

Pathological nodal stage**	**Total (n = 74)**	**Total (n = 89)**	0.747
pN0	33 (44 %)	34 (38 %)	
pN1	19 (26 %)	22 (25 %)	
pN2 or pN3	21 (28 %)	22 (25 %)	
Missing	1 (1 %)	11 (12 %)	

**Histology of the primary tumor**			0.459
Adenocarcinoma	84 (80 %)	407 (84 %)	
Squamous cell carcinoma	21 (20 %)	80 (16 %)	

**Signet ring cell carcinoma**	7 (7 %)	42 (9 %)	0.695

**Differentiation grade**			<0.001
Good-moderate	40 (38 %)	114 (23 %)	
Poor/undifferentiated	46 (44 %)	187 (38 %)	
Missing	19 (18 %)	188 (38 %)	

**Timing of detection**			<0.001
Synchronous	43 (41 %)	372 (77 %)	
Metachronous	62 (59 %)	114 (23 %)	

Median disease-free interval [IQR]**	17 [14,24]	18 [15,27]	0.546

**Location of OMD**			<0.001
Distant organ	83 (79 %)	298 (61 %)	
Brain	32 (30 %)	1 (0 %)	
Lung	15 (14 %)	39 (8 %)	
Bone	12 (11 %)	17 (3 %)	
Liver	10 (10 %)	182 (37 %)	
Soft tissue	8 (8 %)	4 (1 %)	
Other distant organ	6 (6 %)	55 (11 %)	
Extra-regional lymph nodes	13 (12 %)	111 (23 %)	
Peritoneum	9 (9 %)	80 (16 %)	

**Confirmation of OMD**			<0.001
Histology	75 (71 %)	226 (46 %)	
Repeated follow-up imaging	30 (29 %)	263 (54 %)	

* For patients with a resected primary tumor.

** For patients who received resection or definitive chemoradiotherapy of the primary tumor.

Primary tumor treatment consisted of surgery in 74 patients (71%), definitive chemoradiotherapy in 12 patients (12 %), or no primary tumor treatment in 19 patients (17%). Treatment of OMD consisted of local treatment alone in 83 patients (79%), including SBRT alone in 34 patients (33%), metastasectomy alone in 35 patients (32%), or both metastasectomy and SBRT in 14 patients (14%). Local treatment of OMD was combined with systemic therapy in 22 patients (21%), including metastasectomy plus systemic therapy in 14 patients (14%), SBRT plus systemic therapy in 7 patients (7%), or both metastasectomy and SBRT plus systemic therapy in 1 patient (1%). Systemic therapy was predominantly administrated before local treatment of OMD (73%) and generally consisted of 2 chemotherapy agents (68 %). The most common chemotherapy regimen consisted of capecitabine and oxaliplatin (36%, Table 2).

**Table 2:** Treatment characteristics of included patients.

Factor	Local +/− systemic therapy (n = 105)		Systemic therapy only (n = 489)		P-value
**Treatment of primary tumor**					<0.001
Surgery	75	71 %	79	16 %	
Esophagectomy	59	55 %	51	10 %	
Gastrectomy	16	15 %	28	6 %	
Definitive chemoradiotherapy	11	12 %	103	21 %	
No treatment	19	18 %	307	63 %	

**Treatment of OMD**					
Local treatment alone	83	79 %	0	0 %	
SBRT	34	33 %	0	0 %	
Metastasectomy	35	32 %	0	0 %	
Metastasectomy + SBRT	14	14 %	0	0 %	
Systemic therapy plus:	22	21 %	0	0 %	
SBRT	7	7 %	0	0 %	
Metastasectomy	14	14 %	0	0 %	
Metastasectomy + SBRT	1	1 %	0	0 %	
Systemic therapy alone	0	0 %	489	100 %	

**Metastasectomy hospital type (n = 64)**					
Academic hospital	38	60 %	0	0 %	
Non-academic hospital	26	40 %	0	0 %	

**Radiotherapy hospital type (n = 56)**					
Academic hospital	36	64 %	0	0 %	
Non-academic hospital	20	36 %	0	0 %	

			
**Sequencing of systemic therapy (n = 22)**					
Before local treatment for OMD	16	73 %	0	0 %	
After local treatment for OMD	6	27 %	0	0 %	

**Systemic therapy hospital type (n = 489)**					
Academic hospital			78	15 %	
Non-academic hospital			411	85 %	

**First-line systemic therapy**	**Total (n = 22)**		**Total (n = 489)**		
Monotherapy	0	0 %	49	10 %	
Capecitabine	0	0 %	49	10 %	
Doublet	15	68 %	257	53 %	
Capecitabine/oxaliplatin (CapOx)	8	36 %	118	24 %	
Carboplatine/paclitaxel (not for primary tumor)	3	14 %	100	20 %	
5-FU/oxaliplatin (FOLFOX)	2	9 %	39	8 %	
Other	2	10 %	27	6 %	
Triplet	6	27 %	83	17 %	
Epirubicine/oxaliplatine/capecitabine (EOX/EOC)	6	27 %	59	12 %	
Epirubicine/cisplatine/capecitabine (ECC/ECX)	0	0 %	8	2 %	
Docetaxel/oxaliplatine/capecitabine (DOC)	0	0 %	8	2 %	
Epirubicine/cisplatine/5-fluorouracil (ECF)	0	0 %	8	2 %	
Targeted therapy (trastuzumab)	1	1 %	73	15 %	

OMD = oligometastatic disease; SBRT = stereotactic radiotherapy.

A total of 64 patients underwent metastasectomy. Metastasectomy was more commonly applied than SBRT for OMD in the liver (80%), the extra-regional lymph nodes (67%), or the peritoneum (100%). A total of 56 patients underwent SBRT. Applied SBRT schedules are provided in Supplementary File B. SBRT was more often performed than metastasectomy for OMD in the lung (73%) or bone (75%). Local treatment of OMD plus systemic therapy was common in patients with OMD in the liver (50%) or peritoneum (78%, Supplementary File C).

Patients with synchronous as compared with metachronous OMD less often underwent primary tumor resection (47% versus 87%), more often underwent local treatment of OMD plus systemic therapy (37% versus 10%), and had extra-regional lymph node oligometastases (19% versus 2%). Patients with metachronous as compared with synchronous OMD more often underwent local treatment of OMD alone (90% versus 63%) and had brain oligometastases (45% versus 9%, Supplementary File D).

A total of 489 patients who underwent systemic therapy alone for OMD. Patients who underwent systemic therapy alone for OMD more often had gastric cancer (32% versus 15%, p < 0.001), synchronous OMD (77% versus 41%, p < 0.001), liver metastases (37% versus 10%, p < 0.001), and an uncontrolled primary tumor (63% versus 18%, p < 0.001) as compared with patients who underwent local treatment for OMD with or without systemic therapy (Table 1 and Table 2).

**Fig. 2:**
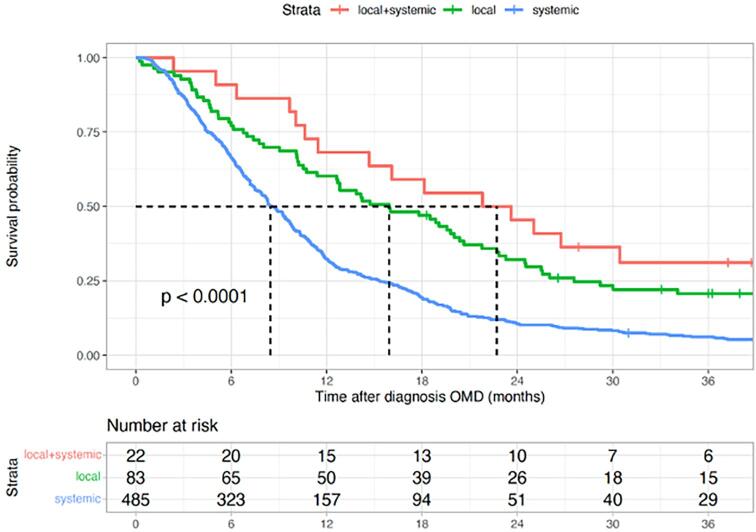
Overall survival curve stratified for treatment of oligometastatic disease.

The median follow-up time for patients undergoing local treatment for OMD with or without systemic therapy was 49.8 months (IQR: 37.2-55.0) and for patients undergoing systemic therapy alone was 59.0 months (IQR: 50.0-62.0). The median OS after local treatment of OMD plus systemic therapy was 22.7 months (95% CI: 14.7-42.6), versus 16.0 months (95% CI: 12.7-21.8) after local treatment of OMD alone, and 8.5 months (95% CI: 7.9-9.6) after systemic therapy alone (Fig. 2).

In multivariable analysis (Table 3), worse OS was independently associated with worse WHO performance scores (HR 1.41, 95% CI: 1.32-1.75; Supplementary File E), poorly or undifferentiated tumor as compared with a good or moderately differentiated tumor (HR 1.37, 95% CI: 1.06-1.76; Supplementary File F), and peritoneal as compared with extra-regional lymph node metastases (HR 1.39, 95% CI: 1.00-1.93; Supplementary File G).

**Table 3:** Results of univariable and multivariable Cox proportional hazard models for overall survival.

		Univariable		Multivariable	
	N =	HR (95 % CI)	*p*-value	HR (95 % CI)	*p*-value
**Age (continuous)**		1.00 (0.99–1.02)	0.079	1.28 (1.00–1.02)	0.018

**Performance score**					
WHO 0	154	Reference	–	Reference	–
WHO > 0	195	1.38 (1.11–1.72)	0.004	1.41 (1.32–1.75)	0.033
Missing	187	1.37 (1.10–1.72)	0.005	1.37 (1.09–1.73)	0.008

**Tumor location**					
Esophagus	328	Reference	–	Reference	–
Stomach	266	1.29 (1.10–1.53)	0.002	0.82 (0.57–1.01)	0.051

**Clinical tumor stage**					
cT1b or cT2	193	Reference	–	Reference	–
cT3	238	1.32 (0.62–0.92)	0.005	0.90 (0.73–1.12)	0.348
cT4	47	0.94 (0.77–1.47)	0.718	1.07 (0.77–1.51)	0.677
Missing	116	0.78 (1.00–1.61)	0.047	1.03 (0.80–1.33)	0.806

**Clinical nodal stage**					
cN0	151	Reference	–	Reference	–
cN1	213	0.78 (0.63–0.97)	0.029	0.80 (0.59–1.00)	0.050
cN2 or cN3	194	0.99 (0.80–1.24)	0.962	0.88 (0.69–1.12)	0.295
Missing	36	1.74 (1.20–2.50)	0.003	1.18 (0.81–1.72)	0.400

**Histology**					
Squamous cell carcinoma	491	Reference	–	Reference	–
Adenocarcinoma	101	1.32 (1.06–1.66)	0.015	1.18 (0.81–1.72)	0.227

**Signet ring cell carcinoma**					
No	545	Reference	–	Reference	–
Yes	49	0.68 (0.51–0.92)	0.011	1.03 (0.94–1.79)	0.170

**Differentiation grade**					
Good-moderate	114	Reference	–	Reference	–
Poor/undifferentiated	187	1.32 (1.04–1.67)	0.022	1.37 (1.06–1.76)	0.015
Missing	293	0.70 (0.56–0.87)	0.002	1.09 (0.85–1.40)	0.479

**Timing of detection**					
Synchronous	415	Reference	–	Reference	–
Metachronous	176	0.95 (0.62–1.46)	0.769	1.06 (0.85–1.32)	0.690

**Location of OMD**					
Extra-regional lymph node	124	Reference	–	Reference	–
Distant organ	320	1.03 (0.83–1.28)	0.791	1.08 (0.85–1.38)	0.529
Peritoneum	129	1.62 (1.26–2.09)	<0.001	1.39 (1.01–1.93)	0.047

**Primary tumor controlled**					
No	505	Reference	ref	Reference	ref
Yes	86	0.78 (0.44–1.36)	0.376	0.48 (0.27–0.86)	0.013

**Treatment for OMD**					
Systemic	486	Reference	–	Reference	–
Local	83	0.32 (0.24–0.41)	<0.001	0.52 (0.31–0.90)	0.018
Local + Systemic	22	0.32 (0.19–0.52)	<0.001	0.42 (0.22–0.82)	0.011

Improved OS was independently associated with local treatment of OMD alone or combined with systemic therapy as compared with systemic therapy alone (HR 0.52, 95% CI: 0.31-0.90 and HR 0.42, 95% CI: 0.22-0.82, respectively), and a controlled primary tumor versus uncontrolled primary tumor (HR 0.48, 95% CI: 0.27-0.86; Supplementary File H).

## Discussion

This nationwide population-based cohort suggests that local treatment of OMD alone or combined with systemic therapy can be a preferred treatment approach for patients with oligometastatic esophagogastric cancer since this treatment approach was independently associated with improved OS as compared with systemic therapy of OMD alone (median OS of 16.0 months or 22.7 months versus 8.5 months). However, these results must be interpreted with care because selection may have resulted in a potential overestimation of OS after local treatment of OMD because patients with favorable patient- and tumor characteristics were more often selected for treatment (i.e. confounding by indication) [33]. In addition, the NCR did not record the number or size of OMD lesions which may have impacted on OS [27]. Therefore, randomized controlled trials are warranted to confirm our results.

The benefit of local treatment of OMD plus systemic therapy over systemic therapy alone has been previously suggested by a phase II non-randomized trial by Al-Batran et al. [14]. This study included patients with gastric or gastroesophageal junction adenocarcinoma with synchronous OMD. Patients who responded to fluorouracil, leucovorin, oxaliplatin, and docetaxel (FLOT) chemotherapy underwent resection of the primary tumor and metastases [14]. This study showed improved OS after resection of the primary tumor and metastases in patients who responded to FLOT chemotherapy as compared with patients who did not respond to systemic therapy (median OS of 31.3 months versus 15.9 months, respectively) [14]. These results have resulted in an ongoing phase III RENAISSANCE trial in which patients with gastric or gastroesophageal junction adenocarcinoma with synchronous OMD who respond to FLOT chemotherapy will be randomized to either continuation of FLOT chemotherapy or resection of the primary tumor and metastases [16]. In addition, the results of our study are comparable with the phase II trial by Liu et al. This study included patients with esophageal squamous cell carcinoma with metachronous OMD who underwent SBRT and 50 % received adjuvant systemic therapy [15]. This study showed an OS of 24.6 months [15]

Although several non-randomized studies have suggested excellent OS in patients undergoing local treatment of OMD plus systemic therapy [14], [15], this study shows that only 21% of patients undergoing local treatment receivedcombined systemic therapy as compared with 100% [14] and 50% [15] in these phase II trials. The limited use of combined local treatment plus systemic therapy in our population-based study was mainly seen in patients with brain oligometastasis, which formed a relatively large proportion of our study population (30%). Chemotherapy has limited activity in the brain, which has been mainly attributed to the blood–brain barrier [34]. Patients with brain oligometastasis were excluded from these phase II trials [14], [15]. Besides the high portion of patients with brain oligometastasis, the limited use of systemic therapy combined with local treatment of OMD might also be explained by the lack of evidence-based guidelines to guide treatment decision-making and the lack of completed RCTs in the setting of esophagogastric OMD.

In addition to the German RENAISSANCE trial, , several phase 3 trials are currently investigating the benefit of local treatment for OMD plus systemic therapy over systemic therapy alone [16], [17], [18]. In the American ECOG study (NCT04248452), patients with synchronous or metachronous OMD limited to 3 metastases will be included [17]. Patients with response to chemotherapy will be randomized to either SBRT plus continuation of chemotherapy or continuation of chemotherapy alone [17]. Finally, in the French SURGIGAST trial (NCT03042169), patients with synchronous gastric cancer with synchronous OMD limited to the retroperitoneal lymph nodes and/or 1 organ with metastases will be included [18]. Patients with response to “standard chemotherapy” will be randomized to either resection of the primary tumor and oligometastases or continuation of chemotherapy [18].

However, none of these studies have incorporated immunotherapy in the treatment algorithm for OMD, although several studies have shown improved survival outcomes for patients with esophagogastric cancer treated with immunotherapy in the first-line palliative setting [35] or in the adjuvant setting after a pathological incomplete response after neoadjuvant chemoradiotherapy and surgery [36]. Currently, it is unknown if immunotherapy also improves survival outcomes in the OMD setting before and/or after local treatment for OMD in patients with esophagogastric cancer. Therefore, a potential future study could assess the benefit of immunotherapy plus local treatment for OMD in patients with esophagogastric cancer.

Certain limitations apply to this study that warrants caution for the interpretation of results. First, no additional prognostic factors could be analyzed in the multivariable Cox proportional hazard model because of the risk of overfitting given the relatively limited sample size [37]. Second, missing data on performance status and differentiation grade may have reduced the power of the current study. Third, no propensity score-matching could be performed due to the limited number of patients in treatment subgroups. However, this is the first population-based cohort study, to the best of our knowledge, on the management and outcomes of local treatment and systemic therapy of esophagogastric OMD. Therefore, this is the first study that provides real-world generalizability and applicability. Other strengths include the register-based follow-up resulting in complete follow-up information for all patients.

The OligoMetastatic Esophagogastric Cancer (OMEC) project aims to achieve consensus on the definition and treatment of oligometastatic esophagogastric cancer (https://www.OMECproject.eu). OMEC is a consortium of 50 esophagogastric cancer expert centers across 16 countries in Europe. Studies of the OMEC-project include a systematic review of definitions of esophagogastric OMD (OMEC-1 [24]), distribution of clinical cases to experts asking for multidisciplinary team responses on diagnosis and treatment (OMEC-2) [38], Delphi consensus through 2 Delphi rounds and a consensus meeting (OMEC-3). The OMEC project will result in a multidisciplinary European consensus statement for oligometastatic esophagogastric cancer (OMEC-4), laying the basis for a prospective clinical study incorporating immunotherapy and local treatment for OMD for these patients (OMEC-5).

## Conclusion

In conclusion, our results suggest that the preferred approach to oligometastatic esophagogastric cancer includes radical local treatment of OMD alone (e.g. metastasectomy or SBRT) or a combined approach consisting of radial local treatment of OMD plus systemic therapy (e.g. chemotherapy) . However, our results are most likely biased. Therefore, randomized controlled trials are warranted to confirm these results.

## Declaration of Competing Interest

The authors declare the following financial interests/personal relationships which may be considered as potential competing interests: Dr. van Laarhoven reports consultant or advisory role: BMS, Dragonfly, Lilly, Merck, Nordic Pharma, Servier; research funding and/or medication supply: Bayer, BMS, Celgene, Janssen, Incyte, Lilly, Merck, Nordic Pharma, Philips, Roche, Servier; Dr. Verhoeven reports grants from Bristol-Myer Squibb and Roche, outside the submitted work; Dr. Haj Mohammad reports personal fees from BMS, Lilly, MSD, Servier, and Astra Zeneca, outside the submitted work; the other authors have nothing to disclose..
