# Supplementary material for: Stereotactic radiotherapy or metastasectomy for oligometastatic esophagogastric cancer: A nationwide population-based cohort study
Source: Clin Transl Radiat Oncol. 2022 Aug 24;37:109–15. doi: 10.1016/j.ctro.2022.08.012 (PMC9523096; doi:10.1016/j.ctro.2022.08.012)

**Supplementary File A**: STROBE checklist

|  | Item No | Recommendation | Included |
| --- | --- | --- | --- |
| Title and abstract | 1 | (*a*) Indicate the study's design with a commonly used term in the title or the abstract | Yes |
|  |  | (*b*) Provide in the abstract an informative and balanced summary of what was done and what was found | Yes |
| Introduction | | | |
| Background/rationale | 2 | Explain the scientific background and rationale for the investigation being reported | Yes |
| Objectives | 3 | State specific objectives, including any prespecified hypotheses | Yes |
| Methods | | | |
| Study design | 4 | Present key elements of study design early in the paper | Yes |
| Setting | 5 | Describe the setting, locations, and relevant dates, including periods of recruitment, exposure, follow-up, and data collection | Yes |
| Participants | 6 | (*a*) Give the eligibility criteria, and the sources and methods of selection of participants. Describe methods of follow-up | Yes |
|  |  | (*b*) For matched studies, give matching criteria and number of exposed and unexposed | NA |
| Variables | 7 | Clearly define all outcomes, exposures, predictors, potential confounders, and effect modifiers. Give diagnostic criteria, if applicable | Yes |
| Data sources/ measurement | 8* | For each variable of interest, give sources of data and details of methods of assessment (measurement). Describe comparability of assessment methods if there is more than one group | Yes |
| Bias | 9 | Describe any efforts to address potential sources of bias | Yes |
| Study size | 10 | Explain how the study size was arrived at | NA |
| Quantitative variables | 11 | Explain how quantitative variables were handled in the analyses. If applicable, describe which groupings were chosen and why | Yes |
| Statistical methods | 12 | (*a*) Describe all statistical methods, including those used to control for confounding | Yes |
|  |  | (*b*) Describe any methods used to examine subgroups and interactions | Yes |
|  |  | (*c*) Explain how missing data were addressed |  |
|  |  | (*d*) If applicable, explain how loss to follow-up was addressed | Yes |
|  |  | (*e*) Describe any sensitivity analyses | NA |
| Results | | |  |
| Participants | 13* | (a) Report numbers of individuals at each stage of study—eg numbers potentially eligible, examined for eligibility, confirmed eligible, included in the study, completing follow-up, and analysed | Yes |
|  |  | (b) Give reasons for non-participation at each stage | Yes |
|  |  | (c) Consider use of a flow diagram | Yes |
| Descriptive data | 14* | (a) Give characteristics of study participants (eg demographic, clinical, social) and information on exposures and potential confounders | Yes |
|  |  | (b) Indicate number of participants with missing data for each variable of interest | Yes |
|  |  | (c) Summarise follow-up time (eg, average and total amount) | Yes |
| Outcome data | 15* | Report numbers of outcome events or summary measures over time | Yes |

| Main results | 16 | (*a*) Give unadjusted estimates and, if applicable, confounder-adjusted estimates and their precision (eg, 95% confidence interval). Make clear which confounders were adjusted for and why they were included | Yes |
| --- | --- | --- | --- |
|  |  | (*b*) Report category boundaries when continuous variables were categorized | Yes |
|  |  | (*c*) If relevant, consider translating estimates of relative risk into absolute risk for a meaningful time period | Yes |
| Other analyses | 17 | Report other analyses done—eg analyses of subgroups and interactions, and sensitivity analyses | NA |
| Discussion | | | |
| Key results | 18 | Summarise key results with reference to study objectives | Yes |
| Limitations | 19 | Discuss limitations of the study, taking into account sources of potential bias or imprecision. Discuss both direction and magnitude of any potential bias | Yes |
| Interpretation | 20 | Give a cautious overall interpretation of results considering objectives, limitations, multiplicity of analyses, results from similar studies, and other relevant evidence | Yes |
| Generalisability | 21 | Discuss the generalisability (external validity) of the study results | Yes |
| Other information | | | |
| Funding | 22 | Give the source of funding and the role of the funders for the present study and, if applicable, for the original study on which the present article is based | Yes |

**Supplementary File B:** Applied SBRT schedules for OMD.

| **Fractions** | **Dosage per fraction** | **Total dosage** | **EQD2 α/β=10** | **BED α/β=10** | **Patients** |
| --- | --- | --- | --- | --- | --- |
| 5 | 7 | 35 | 49.58 | 59.5 | 6 |
| 3 | 10 | 30 | 50 | 60 | 5 |
| 1 | 21 | 21 | 54.25 | 65.1 | 4 |
| 1 | 18 | 18 | 42 | 50.4 | 3 |
| 3 | 8 | 24 | 36 | 43.2 | 3 |
| 3 | 17 | 51 | 114.7 | 137.7 | 3 |
| 1 | 20 | 20 | 50 | 60 | 2 |
| 2 | 12 | 24 | 44 | 52.8 | 2 |
| 1 | 25 | 25 | 72.92 | 87.5 | 2 |
| 3 | 18 | 54 | 126 | 151.2 | 2 |
| 5 | 12 | 60 | 110 | 132 | 2 |
| 3 | 7.5 | 22,5 | 32.81 | 39.38 | 1 |
| 5 | 5 | 25 | 31.25 | 37.5 | 1 |
| 5 | 8 | 40 | 60 | 72 | 1 |
| 3 | 18 | 54 | 126 | 151.2 | 1 |
| 5 | 11 | 55 | 96.25 | 115.5 | 1 |
| 8 | 7.5 | 60 | 87.5 | 105 | 1 |
| 6 | 10 | 60 | 100 | 120 | 1 |
| 3 | 20 | 60 | 150 | 180 | 1 |
| 10 | 7 | 70 | 99.17 | 119 | 1 |
| 12 | 6.25 | 75 | 101.5 | 121.9 | 1 |
| 5 | 20 | 100 | 250 | 300 | 1 |
| 5 | 35 | 175 | 656.2 | 787.5 | 1 |
| NS | NS | NS | NS | NS | 10 |
| EQD2: Equivalent dose in 2Gy fractions; BED: Biological Effective Dose. | | | | | |

**Supplementary File C:** List of treatment modality stratified for the location of OMD

| **Location of OMD** | **Metastasectomy** | | **Metastasectomy + SBRT** | | **SBRT** | | **Local plus systemic therapy** | | **Total** |
| --- | --- | --- | --- | --- | --- | --- | --- | --- | --- |
| **Organ** |  |  |  |  |  |  |  |  |  |
| Brain | 6 | 19% | 10 | 31% | 16 | 50% | 0 | 0% | (n = 32) |
| Lung | 3 | 20% | 1 | 7% | 11 | 73% | 4 | 27% | (n = 15) |
| Bone | 2 | 17% | 1 | 8% | 9 | 75% | 4 | 33% | (n = 12) |
| Liver | 8 | 80% | 0 | 0% | 2 | 20% | 5 | 50% | (n = 10) |
| Soft tissue | 6 | 75% | 2 | 25% | 0 | 0% | 1 | 14% | (n = 8) |
| Other organ | 6 | 100% | 0 | 0% | 0 | 0% | 0 | 0% | (n = 6) |
| **Extra-regional lymph nodes** | 9 | 69% | 1 | 8% | 3 | 23% | 0 | 0% | (n = 13) |
| **Peritoneum** | 9* | 100% | 0 | 0% | 0 | 0% | 7 | 78% | (n = 9) |
| Row percentages; SBRT = stereotactic radiotherapy; * without HIPEC | | | | | | | | | |

**Supplementary File D:** Treatment stratified by synchronous or metachronous OMD

| **Factor** | **Synchronous**  (n = 43) | | **Metachronous**  (n = 62) | |
| --- | --- | --- | --- | --- |
| **Treatment of primary tumor** |  | |  | |
| Surgery | 20 | 47% | 54 | 87% |
| Transthoracic esophagectomy | 9 | 21% | 39 | 63% |
| Total gastrectomy | 5 | 12% | 7 | 11% |
| Transhiatal esophagectomy | 3 | 7% | 7 | 11% |
| Distal gastrectomy | 3 | 7% | 1 | 2% |
| Definitive chemoradiotherapy | 4 | 9% | 8 | 13% |
| No treatment of primary tumor | 18 | 42% | 0 | 0% |
| **Treatment of OMD** |  |  |  |  |
| Local treatment alone | 27 | 63% | 56 | 90% |
| SBRT | 9 | 21% | 25 | 40% |
| Metastasectomy | 16 | 37% | 19 | 30% |
| Metastasectomy + SBRT | 2 | 5% | 12 | 19% |
| Systemic therapy plus: | 16 | 37% | 6 | 10% |
| SBRT | 5 | 12% | 2 | 3% |
| Metastasectomy | 10 | 23% | 4 | 6% |
| Metastasectomy + SBRT | 1 | 2% | 0 | 0% |
| **Location of OMD** |  |  |  |  |
| Organ | 26 | 60% | 58 | 94% |
| Brain | 4 | 9% | 28 | 45% |
| Lung | 7 | 16% | 8 | 13% |
| Bone | 6 | 14% | 6 | 10% |
| Liver | 6 | 14% | 4 | 6% |
| Soft tissue | 1 | 2% | 7 | 11% |
| Other solitary organ | 2 | 5% | 5 | 8% |
| Extra-regional lymph nodes | 10 | 23% | 3 | 5% |
| Peritoneum | 8 | 19% | 1 | 2% |
| SBRT = stereotactic radiation therapy | | | | |

**Supplementary File E:** Overall survival curve stratified by WHO performance score


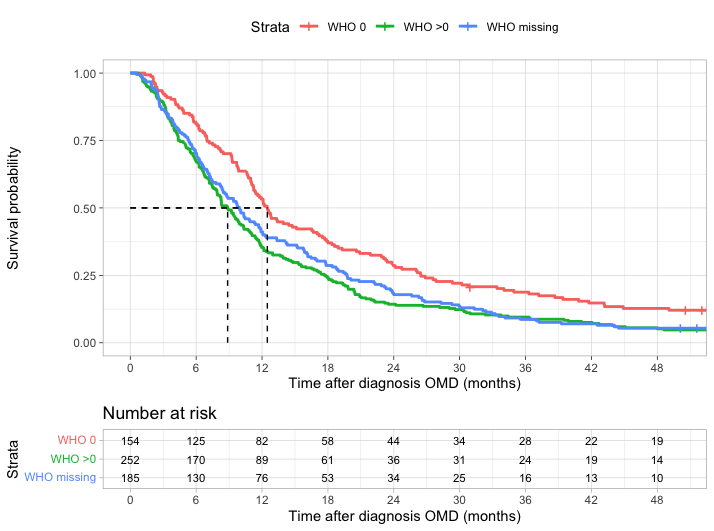


**Supplementary File F:** Overall survival curve stratified by differentiation grade


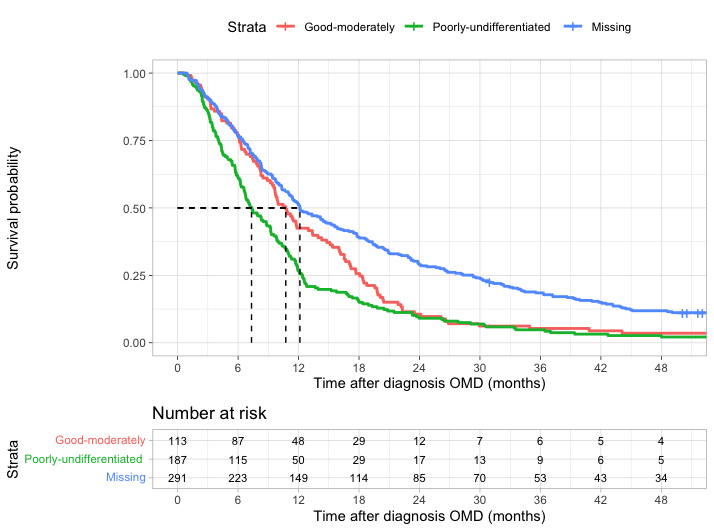


**Supplementary File G:** Overall survival curve stratified by location of OMD


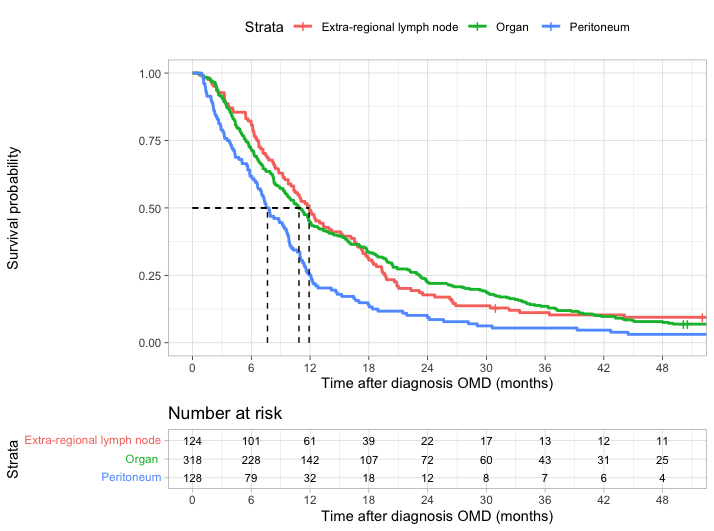


**Supplementary File H:** Overall survival curve stratified by primary tumor treatment


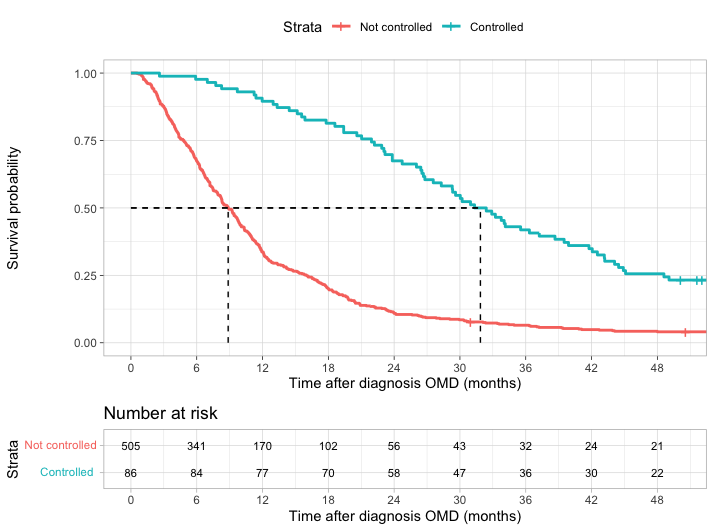

Supplement: Supplementary Data 1 [file mmc1.docx]
